# Measuring the dark triad: a meta-analytical SEM study of two prominent short scales

**DOI:** 10.3389/fpsyg.2024.1469970

**Published:** 2025-01-15

**Authors:** Lukas A. Knitter, Jerome Hoffmann, Michael Eid, Tobias Koch

**Affiliations:** ^1^Department of Psychology, Friedrich-Schiller-Universität, Jena, Germany; ^2^Leibniz Institute for Educational Trajectories (LIfBi), Bamberg, Germany; ^3^Department of Education and Psychology, Freie Universität, Berlin, Germany

**Keywords:** dark triad (DT), bifactor model, TSSEM, meta-analysis, measurement model

## Abstract

This research examines the factor structure and psychometric properties of two well-known Dark Triad personality trait questionnaires: the Short Dark Triad (SD3) and the Dirty Dozen (DD). By analyzing data from 11 (SD3) and 5 (DD) carefully selected studies in the United States and Canada, this meta-analysis uncovers unexpected correlations among questionnaire items, challenging existing assumptions. The study employs a two-stage structural equation modeling approach to evaluate various measurement models. Conventional models, such as the correlated factor and orthogonal bifactor models, fail to explain the irregular correlations. For Dirty Dozen items, a bifactor-(S·I-1) model is more suitable than the orthogonal bifactor model, significantly affecting interpretation. On the other hand, the complex structure of the SD3 necessitates item revision to enhance reliability, discriminant validity, and predictive validity. These findings emphasize the need for refining and clarifying concepts in item revision. Furthermore, the research highlights the overlap between Machiavellianism and psychopathy, particularly in relation to revenge-related items, suggesting the need for differentiation between these traits or the identification of distinct core characteristics.

## 1 Introduction

The Dark Triad (DT), comprising narcissism, psychopathy, and Machiavellianism, has long captivated the field of psychology as a construct representing non-pathological, non-forensic malignant personality traits. The conceptualization of DT can be traced back to Paulhus and Williams (2002), who defined it as an overarching construct characterized by self-aggrandizement, emotional coldness, insincerity, and aggressiveness. Recent findings further highlight central elements of the DT traits, including callousness, primary psychopathy, Machiavellianism, pathological selfishness, and narcissistic rivalry (Dinić et al., 2021; Dinić et al., 2020). In addition, each trait has unique features, such as superiority in narcissism, impulsivity and low empathy in psychopathy, and manipulative tendencies and cynicism in Machiavellianism (Dragostinov and Mõttus, 2022; Paulhus and Williams, 2002).

**Figure 1:**
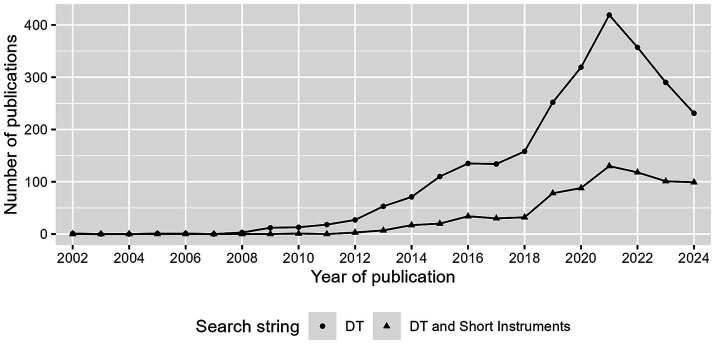
Number of publications per year. Search in web of science (on 30/10/2024) with search string DT: “Dark Triad” AND “Personality,” and DT and Short Instrument: (“SD3” OR “Short Dark Triad” OR “Dirty Dozen” OR “DTDD”) AND “dark triad” OR “dark tetrad”.

Over the past two decades, the measurement of the DT has received considerable attention, as evidenced by the exponential growth in annual publications, as shown in Figure 1. The Dirty Dozen (DD) questionnaire (Jonason and Webster, 2010) and the Short Dark Triad (SD3) questionnaire (Jones and Paulhus, 2014) have emerged as the most widely used short DT questionnaires. Since 2022, the popularity of the DT as a whole seems to be declining. However, publications on the DD and SD3 are still popular, with approximately 100 studies published in recent years (see Figure 1). The rapid application of these measures has facilitated numerous insights into the DT, exploring its associations with other personality traits (Kowalski et al., 2019; Schreiber and Marcus, 2020), athletic experience (Vaughan et al., 2019), sociosexuality (Garcia, 2020), and work performance (O'Boyle et al., 2012). Given its social, political, and academic relevance, the measurement of DT has received increasing attention.

The psychometric evaluation of DT questionnaires is crucial as their quality directly affects research validity and reliability. This study systematically compares competing measurement models using a meta-analytic structural modeling approach and provides new insights into the assessment of DT. To our knowledge, this is the first meta-analytic comparison of its kind.

### 1.1 The rise of the dark triad

The DT questionnaires have become popular because it is easier to include all three traits in one survey with only 27 or 12 items, compared to other measures that have up to 124 items (Muris et al., 2017). The substantial correlations between the three constructs led to the conceptualization of the triad but also to a discussion about whether the traits are interchangeable in the normal population (e.g., Muris et al., 2017, correlation between narcissism and psychopathy: $r_{NP}=.38$ , between Machiavellianism and narcissism: $r_{MN}=.34$ , between Machiavellianism and psychopathy: $r_{MP}=.58$ ). Comparisons with traits from established personality models, the Five-Factor Model (FFM) and the HEXACO model, showed that these did not fully account for the DT traits (Schreiber and Marcus, 2020). Furthermore, the DT traits each showed different patterns of correlation. Supplementary Table 1 shows significant correlations between the DT traits and other established personality traits reported in three recent meta-analyses.

#### 1.1.1 Criticism of DT measurement

The distinction between Machiavellianism and psychopathy has always been highly criticized across measures (e.g., Kowalski et al., 2021; McHoskey et al., 1998). Even though they seem to be theoretically different, it cannot be shown empirically (Vize et al., 2018). Miller et al. (2017) examined the distinctiveness of the two constructs based on several questionnaires. Their results showed that the factors shared nearly 80% of the variance, raising the question of what remains trait-specific. This is supported by meta-analytic evidence, in which the authors agree that measures of Machiavellianism do indeed measure psychopathy (Vize et al., 2018). On the other hand, there are many studies that argue both theoretically and empirically in favor of differentiability (Jones and Paulhus, 2010; Kowalski et al., 2019). This is based on correlations with the facets of the FFM. Reference is made to the high degree of similarity between the profiles but also to the existing—albeit small—differences (Kowalski et al., 2019). Proponents of indistinguishability, on the other hand, argue that the almost identical FFM profiles, despite minor differences, speak for their redundancy (O'Boyle et al., 2015). This raises a fundamental question in personality psychology: How much difference is necessary for traits to be considered different? It should be noted that establishing distinctiveness was not deemed necessary to define a dark trait (Kowalski et al., 2021).

Even beyond personality models, there is evidence that shows that Machiavellianism and psychopathy are related to different external criteria, such as cheating behavior (e.g., Jones et al., 2021) and impulsivity (e.g., Malesza and Kalinowski, 2019). The problem with this evidence is that the correlations of the traits with external criteria seem to depend on the instruments chosen (Schreiber and Marcus, 2020; Watts et al., 2017). There may be several reasons for this. On the one hand, different authors may have different ideas about the constructs, and on the other hand, there may be a lack of consistency in the instruments. Miller et al. (2019) argue that Machiavellianism is generally mismeasured and recommend revising the measurement of the Machiavellianism construct itself. According to them, none of the measures of the Machiavellianism scale matched expert descriptions of the construct. Thus, despite the theoretical distinctiveness of the constructs, the instruments are not well grounded in this theory.

This is particularly problematic because some of the conceptual features of these traits are diametrically opposed: Psychopathy is said to be related to short-term gains, whereas Machiavellianism is related to long-term gains (Furnham et al., 2013). Whereas psychopathy is characterized by high impulsivity, Machiavellianism is said to be characterized by high self-control (Vize et al., 2018). The lack of differentiability is reflected in the measures. Machiavellianism, as already mentioned, is associated with low conscientiousness, which contradicts the theory (Miller et al., 2019). Another problem that arises in the tradition of the DT is the neglect of the multidimensional structure of the constructs. Whereas the single-construct literature takes account of the multidimensional structure of psychopathy, narcissism, and, more recently, Machiavellianism, the DT literature and instruments largely ignore this fact (Miller et al., 2019). Both the SD3 and the DD provide only one score per construct (Jonason and Webster, 2010; Jones and Paulhus, 2014).

In response to this criticism, three main strategies can be found in the current literature: reducing the number of dark traits (Garcia and Rosenberg, 2016; Persson et al., 2019; Sharpe et al., 2021), including additional dark traits (Buckels et al., 2013; Moshagen et al., 2018; Paulhus et al., 2021), and maintaining the number of traits while improving the items or the selection (Krasko and Kaiser, 2023; Küfner et al., 2014). A fourth strategy: developing a new questionnaire that addresses the root of the problem is rarely found in the literature. As far as we know, the work of Paulhus et al. (2021) is the only one to date.

All these approaches have in common that they have taken the criticism of psychometric properties as a starting point but have not—or not fully—addressed it. An important limitation of reducing the DT to a dyad is that it does not necessarily improve the fit of the simple structure (Persson et al., 2019). Removing bad items from existing measures is a common and effective approach to improve measurement quality. However, this can lead to a loss of reliability and validity of the measure and can introduce error and bias in the assessment of DT traits. The most promising approach is to replace problematic scales completely if necessary. Developing the SD3 into the Short Dark Tetrad Scale (SD4), Paulhus et al. (2021) added sadism as a fourth trait and selected new Machiavellian items to reduce the often-criticized overlap. Initial investigations indicate that this has been successful. The correlated factor model shows that Machiavellianism and psychopathy share between 18 and 24% of their variance (Neumann et al., 2022; Paulhus et al., 2021). However, there is a high degree of overlap between psychopathy and sadism (38 and 45% shared variance). Blötner and Beisemann (2022) attribute the problems to the SD4 sadism scale. In the Serbian adaptation, however, the SD4 psychopathy scale seems to suffer from validity problems (Dinić et al., 2024). Furthermore, expanding the DT to include additional traits may lead to conceptual confusion and lack of clarity about what is being measured (Sleep et al., 2017). Although SD4 seems to have successfully addressed the problematic overlap between Machiavellianism and psychopathy, it is not yet as established as DD and SD3. The latter are still widely used, which is why this study focuses on them.

### 1.2 Two prominent short questionnaires

The DD (Jonason and Webster, 2010) and SD3 (Jones and Paulhus, 2014) questionnaires each consist of three scales representing different characteristics. The DD has four items per scale (12 items in total), while the SD3 has nine items per scale (27 items in total). For the DD, single-construct instruments were used as the initial item pool, and in the end, 12 items were selected based on their centrality to each trait using principal component analysis (PCA) (Jonason and Webster, 2010). The final DD showed moderate correlations between the three extracted factors. The internal consistency is relatively high for the total scale ( α=.83 ) and low to moderately high at the scale level: between 0.44 and 0.64 for psychopathy, 0.81 and 0.87 for Machiavellianism, and 0.81 to 0.88 for narcissism. Recent studies had reported higher coefficient alphas for all scales ( α=.71 for psychopathy, α=.85 for Machiavellianism, and α=.80 for narcissism, Dragostinov and Mõttus, 2022).

In contrast, the construction of the SD3 (Jones and Paulhus, 2014) was based on theoretical foundations. A 41-item pool was created, and through several steps of analysis, including PCA and exploratory factor analysis, the initial items were reduced to a final set of 27 items. The SD3 showed moderate coefficient alphas: 0.71 to 0.76 for Machiavellianism, 0.72 to 0.77 for psychopathy, and 0.68 to 0.78 for narcissism. Similar findings have been reported in recent studies (Dragostinov and Mõttus, 2022; McLarnon and Tarraf, 2021; Sleep et al., 2017).

Both the SD3 and DD have been criticized for low discriminant validity between traits, as shown by multitrait-multimethod analyses (Jonason and Webster, 2010; Jones and Paulhus, 2014). Machiavellianism and psychopathy in both questionnaires are highly correlated with single-construct instruments for both constructs, while narcissism is sufficiently discriminated from other scales (Siddiqi et al., 2020). Furthermore, the DD was found to be too short and to capture smaller proportions of variance in the established single-construct scales (Maples et al., 2014; Miller et al., 2012). The SD3 captures more variance of the established scales in all scales (Maples et al., 2014). However, it should be noted that the authors did not consider vulnerability as part of subclinical narcissism, and the SD3 does not capture it (Jones and Paulhus, 2014). Instead it focus on grandiosity, which is more relevant in the DT research (Furnham et al., 2013). However, both questionnaires have attempted to break down multidimensional constructs into single dimensions (Miller et al., 2019), and their brevity and consequent economy is their most obvious advantage.

### 1.3 Factor structure of the dark triad

**Figure 2:**
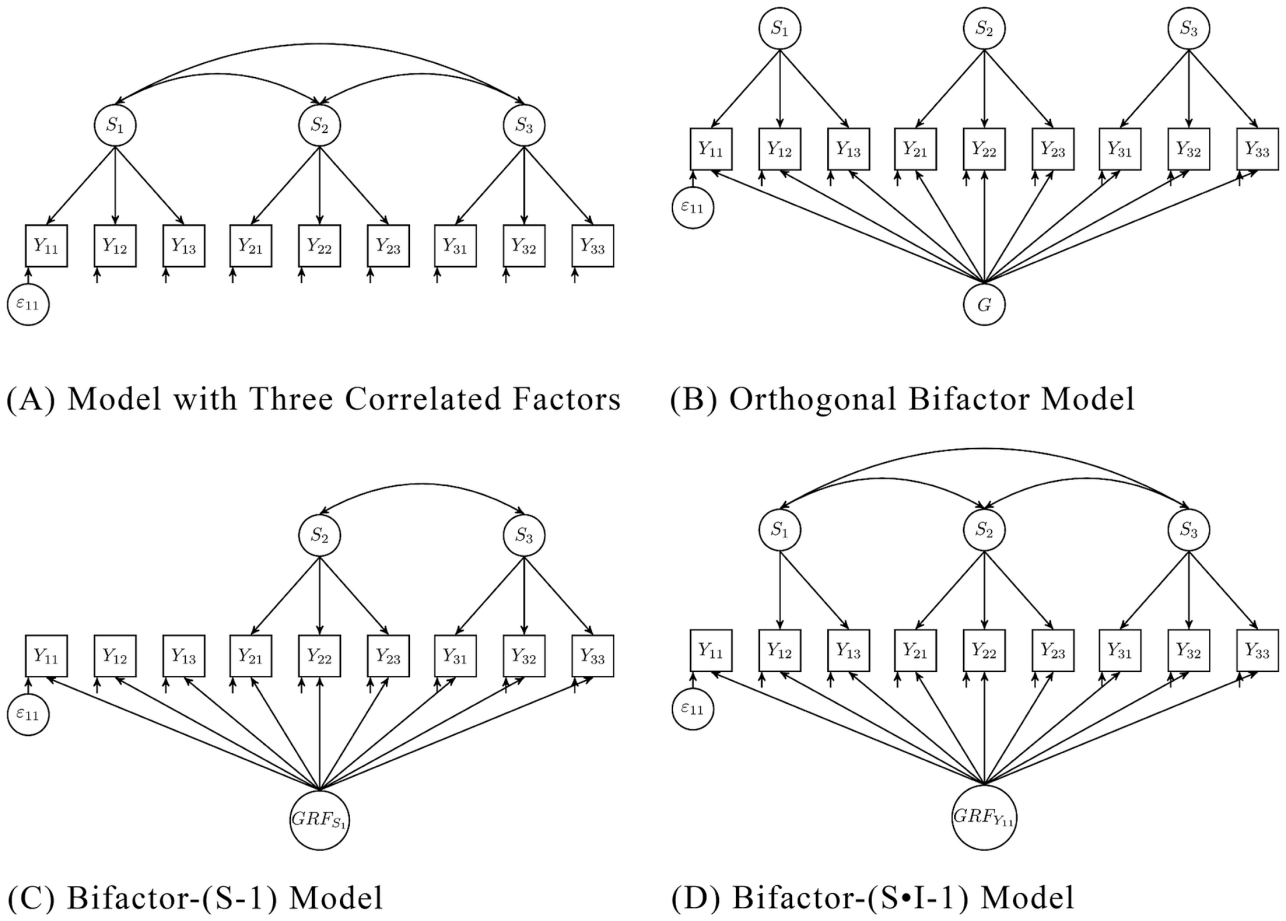
**(A)** Model with three correlated factors, **(B)** Orthogonal Bifactor Model, **(C)** Bifactor-(S-1) Model, **(D)** Bifactor-(S·I-1) model. S_i_ = Specific Factor i, Y_ij_ = Item j of Factor i, ε_ij_ = Residuum of Item Y_ij_, G = General Factor, GRF_S1_ = General reference factor, with Factor S_1_ as reference, GRFY_11_ = General reference factor, with Item Y_11_ as reference.

Using confirmatory factor analysis, several measurement models were fitted to SD3 and DD data. Among the commonly used models are the single-factor model, correlated three-factor model (see Figure 2A), and the orthogonal bifactor model with three specific factors (see Figure 2B; Holzinger and Swineford, 1937). The orthogonal bifactor model has often been identified as a model with superior fit in previous studies (e.g., Jonason and Luévano, 2013; McLarnon and Tarraf, 2017; Vaughan et al., 2019). It decomposes each item into three parts: a general factor, a specific factor, and a residual variable (Holzinger and Swineford, 1937). All factors and residuals are uncorrelated. The general factor represents the common variance of all items or indicators. Specific factors are the variance common to a subset of indicators when the variance shared by all is removed. The residuals represent the variance not shared with other items. The general factor was often interpreted as the common trait underlying all measured characteristics. The specific factors were interpreted as if they represent unique characteristics of each trait—such as impulsivity for psychopathy, manipulativeness for Machiavellianism, or grandiosity for narcissism—after accounting for the shared general factor (McLarnon and Tarraf, 2017; Moshagen et al., 2018). They are often referred to only as Machiavellianism, psychopathy, and narcissism (e.g., McLarnon and Tarraf, 2017; Vaughan et al., 2019). While this model seems appealing because it fits the idea of a “dark core,” it is not without problems, leading to ongoing debates in psychometrics.

#### 1.3.1 Issues with the orthogonal bifactor model

One of the key issues with the traditional orthogonal bifactor model is its assumption of interchangeability of modeled facets (Eid et al., 2017). Interchangeability means that the different facets modeled in a bifactor model can be considered a random sample from a universe of facets being equally appropriate to measure an underlying disposition. For example, to measure a narcissism disposition, a sample of social situations can be drawn from a potential universe of social situations, the narcissistic behavior can be measured by multiple items in each situation, and the data can be modeled by a bifactor model. In this case, the general factor would measure the disposition to behave in a narcissistic way (across situations), and a specific factor would represent deviations due to specific qualities of a social situation (not shared with other situations). The social situations would be interchangeable because they are all social stimuli to elicit the disposition of narcissism, and for measuring the narcissism disposition, it is not important which specific social situation were considered if there is a sufficiently large sample of situations being capable to elicit the disposition. However, the DT traits are not randomly selected from a universe of facets of a general trait.

It is important to note that Paulhus and Williams (2002) have selected the three traits for theoretical reasons because they are partially overlapping but also distinct. Two traits overlap in very specific ways, but this overlap can be different from the overlap between two other traits. It is also important to note that Paulhus and Williams (2002) defined a triadic model (see Figure 1) but not a model with a general factor. After an overview of the relationships between the traits, they conclude that they are neither “equivalent” (Paulhus and Williams, 2002, p. 562) nor “interchangeable” (Furnham et al., 2013, p. 204). For these substantive theoretical reasons and for the measurement theoretical reasons described above, the bifactor model is not appropriate. Therefore, it is not amazing that the application of the bifactor model to the DT traits revealed problematic and unrealistic results, such as factor loadings that are close to zero, negative, or insignificant (e.g., Jonason and Luévano, 2013; Persson et al., 2019). Such anomalies challenge the theoretical conception of the model and can result in changes in the meaning of the factors across different samples (Eid et al., 2017; Markon, 2019). Moreover, parameter estimation may be less accurate, leading to negative variance estimates or convergence problems (for DT data, e.g., Rogoza et al., 2021).

The inappropriateness of the bifactor model does not mean that there are not common causes of the traits. According to Furnham et al. (2013, p. 204) “among the strongest candidates are disagreeableness, honesty-humility, lack of empathy (callousness), and interpersonally antagonism.” Recent evidence supports antagonism, especially its facet callousness (Dinić et al., 2021). To analyze the latent common core of the Dark Triad traits, these candidate traits should be included directly as measured indicators of a latent construct, with the three dark traits modeled as dependent outcomes within a structural framework. This could be done by a bifactor-(S-1) model (see Eid, 2020; Eid et al., 2017). This model is conceptually different from the bifactor model and well defined on measurement theory for this type of application. The fact that the bifactor model is inappropriate for analyzing the DT does also not mean that the three traits should not be integrated in a single score. This might be meaningful in different contexts.

#### 1.3.2 Alternative bifactor models

Alternative bifactor models have already been proposed to address the criticisms of the original orthogonal bifactor model (Eid, 2020; Eid et al., 2017; Koch and Eid, 2023). For instance, the bifactor-(S·I-1) model (see Figure 2D) has been suggested. This model has a general factor that loads on all indicators, and specific factors that load on only a subset of items representing a scale. Unlike the orthogonal bifactor model, one item is specified as the reference for the general factor, and the correlations between the specific factors are freely estimated. Depending on the scaling of the factor, either the loading of this indicator or the variance of the general factors is set to one. Specifying a reference changes the meaning of all factors, compared to the orthogonal bifactor model. In the bifactor-(S·I-1) model, the general reference factor (GRF) represents the variance that all items share with the reference item, while the scale-specific factors represent the variance that is not shared with the reference but with a subset of items.

Less technically, we can say that the GRF factor is the latent variable that includes the characteristics that all items share with the reference item. Accordingly, the specific variables combine characteristics that they share with each other but not with the reference (e.g., narcissistic characteristics that are not shared by all Machiavellianism items). In the bifactor-(S·I-1), the correlations between the specific factors can be due to characteristics that are shared between the non-reference scales but not with the reference (e.g., narcissistic characteristics that are shared with psychopathy but not with the Machiavellianism item). The bifactor-(S·I-1) model is specifically designed for scales with less homogeneous intrascale correlations. Therefore, it includes an additional factor for all non-reference items of the same scale as the reference item. It can capture traits that are part of the scale (e.g., Machiavellianism) but are not captured by the selected reference item (e.g., a selected Machiavellianism item).

Another alternative model is the bifactor-(S-1) model (see Figure 2C), which is similar to the previous model. It differs in that an entire scale is specified as the reference, giving the GRF meaning accordingly. The respective specific factor is omitted. It is therefore more appropriate for scales with homogeneous intrascale correlations. The remaining scale-specific factors represent the variance that is not shared with the reference but with the respective subset of items.

Or, to put it less technically, the GRF factor is the latent variable that contains the characteristics that all items share with the reference scale. Accordingly, the specific variables combine characteristics that they share with each other but not with the reference (e.g., narcissistic characteristics that are not shared with all Machiavellianism items). In the bifactor-(S-1) model, the correlations between the specific factors can be due to characteristics that are shared between the non-reference scales but not with the reference (e.g., narcissistic characteristics that are shared with psychopathy but not with Machiavellianism).

It is important to note that the choice of reference has a direct influence on the meaning of the factors, the level of the respective loadings, and thus on the model fit. This is also true for the orthogonal bifactor model if the aforementioned interchangeability assumption is not met, with the difference that the reference and the meaning of the factors are not determined *a priori* but are assigned by the algorithm on the basis of the sample data. Therefore, the fit is often optimized for a given sample. However, the meaning may vary in different samples depending on the data-driven reference (Eid et al., 2017).

In the analysis of SD3 data, a bifactor-(S·I-1) model has been applied on a subset of items (Wehner et al., 2021). However, the critical aspect of reference selection has not been adequately addressed. The choice of reference influences the meaning of the factors and should depend on the specific problem being investigated and the underlying theory. In the absence of explicit concepts, utilizing the intercorrelations among the items or the known loading structure of other measurement models can provide valuable guidance for this selection process. For example, items that tend to be highly correlated with all other items or that show a high loading on a general factor in an orthogonal bifactor model may be good candidates for the reference.

### 1.4 Summary of the issues and present research

The problems with psychometrics and measurement models found in the DT, SD3, and DD literature can be summarized as follows:

- Low discriminant validity between DT traits (O'Boyle et al., 2015; Vize et al., 2018),
- Neglect of the multidimensional structure of DT traits (Miller et al., 2019),
- Problems with the interchangeability assumption of the orthogonal bifactor model (Eid et al., 2017),
- Overfitting issues with the orthogonal bifactor model (Bonifay and Cai, 2017).

These limitations highlight the need for further refinement and development in the measurement of DT traits and the selection of appropriate measurement models. Although a two-factor solution, reflecting the overlap between psychopathy and Machiavellianism, has been suggested (e.g., Persson et al., 2019), the three-factor solution remains more popular and widely utilized in instrument development.

The present study focuses on examining the structure of the SD3 and DD. We focused on studies conducted within English-speaking North American, non-clinical, and non-forensic contexts, published between 2019 and July 2021. This relatively narrow scope was chosen to enhance internal validity and ensure data quality. Limiting the geographical and cultural context minimizes language and cultural confounds, while excluding clinical and forensic samples reduces variability from extraneous influences. Based on this, we want to address the following research questions:

*Research Question 1*: Are existing short questionnaires suitable for reliably and validly measuring Dark Triad personality traits?

*Research Question 2*: What is a meaningful measurement model underlying the questionnaires?

*Research Question 3*: Is the traditional orthogonal bifactor model appropriate for assessing Dark Triad personality data, with existing short questionnaires?

By addressing this questions, we aim to provide new insights into the structural validity of the SD3 and DD. Specifically, this study extends prior work by applying in-depth psychometric analyses that include the examination of item-level metrics, such as interscale and intrascale mean inter-item correlations (MIC), and factor-level metrics, such as the average variance extracted (AVE) and shared variance between factors to assess convergent and discriminant validity. Furthermore, we calculate congeneric, instead of tau-equivalent (‘coefficient alpha’) reliability. In addition, we evaluate the appropriateness of the traditional orthogonal bifactor model for DT traits by critically considering its underlying assumptions and potential limitations. Finally, we explore alternative measurement models to better account for the multidimensionality and the overlap between DT traits, thereby offering a deeper understanding of their latent structure. This approach addresses gaps in the existing literature and offers practical recommendations for future instrument refinement and selection of measurement models.

## 2 Methods

### 2.1 Literature search and data acquisition

At the start of the research, Schreiber and Marcus (2020) was the last published meta-analysis with DT data, which included data up to the end of 2018. In their study, they focused on the location of the DT in other personality models. We focus on studies published from 2019 onwards in our literature search, which was conducted at the end of July 2021. This relatively short time window for a meta-analysis was chosen to minimize possible sociohistorical influence and proved sufficient to identify hundreds of potentially suitable studies.

**Figure 3:**
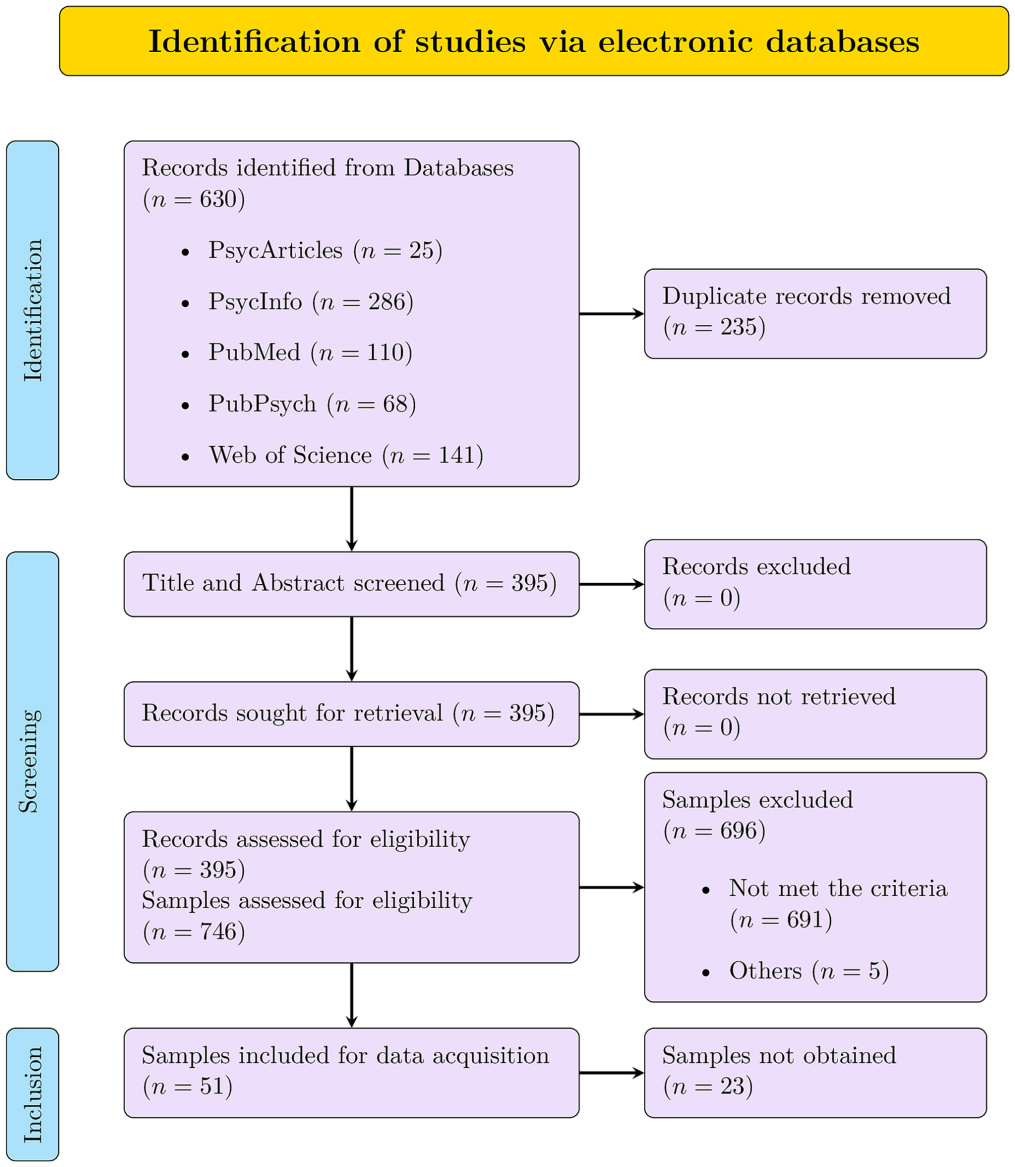
Preferred reporting items for systematic reviews and meta-analyses (PRISMA) flowchart of search procedure.

The flowchart illustrating the data collection process is shown in Figure 3. The following databases were searched: PsycArticles, PsycInfo, PubMed, PubPsych, and Web of Science Core Collection. The search string was (“SD3” OR “Short Dark Triad” OR “Dirty Dozen” OR “DD”) AND “dark triad” OR “dark tetrad” OR (“narcissism” AND “Machiavellianism” AND “psychopathy”). After removing duplicates, 395 studies remained. We were able to retrieve all of them. The review of the articles was divided equally (as far as possible) between four reviewers: The first and second authors and two student assistants.

They rated the articles, following predefined inclusion criteria: (a) at least one scale of the brief measures (DD, SD3) was used, (b) no review or meta-analysis, (c) sample’s country of origin was USA or English-speaking part of Canada, (d) no clinical or forensic samples, (e) participants minimum age of 18, (f) DD or SD3 was assessed as self-report, (g) sample size of at least 180 (for DD) or 405 (for SD3). Simulation studies suggest that the optimal sample size is in the range of 5 to 10 times more participants than the number of free parameters (Bentler and Chou, 1987). The orthogonal bifactor model was decisive as it was the model with the most free parameters (SD3: 81, DD: 36). The decision to include only English-speaking North American, non-clinical, and non-forensic samples in the meta-analysis is justified for several reasons. By adhering to this selection criterion, the meta-analysis benefits from increased internal validity by eliminating language and cultural confounds. Although the results reported here can only be related to this group of individuals, they are more precise and benefit from higher data quality. The exclusion of clinical and forensic samples also helps to minimize confounding variables, ensuring that the results accurately reflect the intended constructs.

Not every study included a specific minimum age requirement, but when Amazon’s Mechanical Turk (MTurk) was used, we still considered the criterion to be met as only people of proven legal age could work on the platform. We also decided to code a less strict criterion, a mean age of at least 18 years. We coded less stringent versions of criteria (c), French-speaking part of Canada, and (g) sample size of at least 200 for SD3 but ultimately did not include them in the final analysis. The stricter initial criteria provided a sufficient dataset to draw accurate and reliable conclusions, making the inclusion of these studies unnecessary.

In addition, we only included articles published in English. To ensure that every rater proceeds in the same way, the first author created a guideline for the coding process (see OSF repository).^1^ Related to the sample size all data ultimately used met the strict requirements. The criteria were specified in such a way that they left little room for interpretation; therefore, no inter-rater correlations were calculated. In case of ambiguity, the first and second author consulted and jointly decided whether a study met the criteria. In addition to coding whether the conditions were met, concrete information was also recorded. These are the sample size, the country of origin of the sample, the minimum, maximum and average ages, any instruments used to collect DT data, the number of items used, and the scale used to answer the items.

We identified 22 relevant samples for the DD and 30 for the SD3. Subsequently, an attempt was made to obtain the data of these. We focus on getting the data for the respective questionnaire on item-level or the covariance or correlation matrix, together with sample size. To maximize these results, we did not include any covariates or descriptive characteristics. We searched the Supplementary material of the articles, repositories, and author websites for the data. If the data could not be found online, the corresponding authors were contacted and asked for the data. In the end, we received 11 unique samples, out of 10 publications, for the DD and 16 unique samples, described in 14 articles, for the SD3. We excluded additional five samples for the DD and three for the SD3 because their correlation matrices clearly differ from the others (see section: Result Evaluation). This means that the correlations between the items in these samples will strongly differ from the corresponding correlations in the majority of the samples. To ensure a valid comparison, we did not pool the above samples as they were significantly different from the other samples. Not fitting models to an inhomogeneous pooled matrix was also recommended by Cheung (2015a).

### 2.2 Data analysis

All analyses were performed in R (version 4.1.2, R Core Team, 2021). To impute missing values, we used the packages *mice* (van Buuren and Groothuis-Oudshoorn, 2011) and *mitools* (Lumley, 2019). Meta-analytic structural equation modeling (MASEM) was estimated using the *metaSEM* package (Cheung, 2015b). Other useful R packages that have been used on a smaller scale can be found in the R scripts in the OSF repository.

Prior to the meta-analytical SEM analysis, the data were prepared as follows. If there were no DT data for a subject, those records were excluded. If individual responses were missing, we imputed the values using predictive mean matching. To do this, 3 to 5 imputations are often performed (Rubin, 1987), although a higher number of imputations are recommended (Graham et al., 2007; von Hippel, 2009). In our study, we performed 10 imputations per dataset to properly address missing data in the analysis. A higher number of imputations (20) did not change the results. We used only data collected by using the original, final items published in the appendix of Jones and Paulhus (2014) for the SD3 and in Table 8 of Jonason and Webster (2010) for the DD. In some studies, some SD3 items were replaced with alternative versions. In these cases, responses to these items were excluded from further analysis. Where necessary, responses were reverse coded. Inter-item correlation matrices were then calculated for each dataset.

For our meta-analysis, we used two-stage structural equation modeling (TSSEM), a meta-analytic approach to structural equation modeling (Cheung, 2015a). In the first stage of TSSEM, correlation matrices were pooled using multiple group SEM with maximum likelihood estimation. In the second stage, measurement models were fitted using weighted least squares estimation. This estimator accounts for missing correlations across samples and allows for more accurate estimation. For further details, see Cheung (2015a). In TSSEM, it is possible to calculate both random effects models (REM) and fixed effects models (FEM). We chose FEM for our analyses because we had strict requirements regarding the origin of the samples to ensure homogeneity.

In the second stage of the TSSEM, we applied six measurement models for the two short questionnaires: a model with three correlated first-order factors, an orthogonal bifactor model, three bifactor-(S-1) models (each factor is considered once as a reference), and a bifactor-(S·I-1) model. For the model with three correlated first-order factors, we specified one factor per DT scale (Machiavellianism, narcissism, psychopathy), each measured by four items (DD) or nine items (SD3). They could be correlated. For the orthogonal bifactor model, in addition to the three specific factors, we specified a general factor D. In this case, the specific factors did not correlate with each other or with D. For the bifactor-(S-1) model, we specified one factor each as reference, called $GRF_{\mathrm{Mach}}$ (Machiavellianism as reference), $GRF_{\mathrm{Narc}}$ (Narcissism as reference), and $GRF_{\mathrm{Psyc}}$ (Psychopathy as reference). In the bifactor-(S·I-1) model, we had chosen item 4 (DD) and item 24 (SD3) as reference.

For the DD, Kajonius et al. (2016) showed that item 4 is a very good representation of the overall scale. This is also consistent with our results for the bifactor model (see section: DD Coefficients). For SD3, there is no theoretical or empirical evidence in the literature to suggest a potential reference candidate. Therefore, we rely on the results of the orthogonal bifactor model (see section: SD3 Coefficients). If an item’s loading on the general factor is high, while the loading on the associated specific factor is close to zero, this is an indicator that this item is a good reference. In both alternative bifactor models, the correlations between the scale-specific factors were freely estimated. In all models, we restricted the variance of the factors to one and report standardized coefficients.

For the application of FEM in TSSEM, the homogeneity of the sample matrices is important. We checked this using test statistics, the root mean square error of approximation (RMSEA), and the standardized root mean squared residual (SRMR). According to Cheung (2015a), the Comparative Fit Index (CFI) and the Tucker–Lewis Index (TLI) are not appropriate measures for this purpose. At present, there is no published research that has verified appropriate cutoff values of RMSEA and SRMR for homogeneity tests in TSSEM stage one. Therefore, the established rules for good SEM model fit of Hu and Bentler (1999) were followed. We assumed homogeneity when RMSEA≤.06 and SRMR≤.08 . The chi-square test statistic could be used in stage one to test the hypothesis that all covariance matrices are equal. However, with large samples ( N≥300 ), even small deviations can lead to a rejection of the hypothesis (Kline, 2023), so we did not consider it.

In case of heterogeneity, we modified the included samples. To do this, we calculated the standardized deviation (SRMR) from the first pooled matrix for each sample. The usual evaluation of the SRMR is that a value less than .08 indicates a good fit, a value between .08 and .10 is often described as acceptable, and a value greater than .10 stands for a poor fit (Kline, 2023). For the evaluation of divergent samples, we adopted a modified approach to the evaluation of SRMR. To account for the pooling of correlation matrices, we set a cutoff for the SRMR that was intentionally higher than the traditional threshold. The rationale behind this decision was to account for the expected lower deviation of individual sample matrices from the pooled matrix when most of the divergent matrices are excluded. In this way, we aimed to exclude all potentially divergent samples while ensuring that as many samples as possible were retained for our analysis. This method allowed us to strike a balance between retaining an adequate number of samples and filtering out outliers that could have skewed our results. We excluded samples with SRMR≥.12 . This value is based on the range of our SRMR values. It is not based on nor is it a general recommendation. After exclusion, a new pooled matrix of the remaining studies was computed. All included individual matrices have an SRMR≤.10 from this new pooled matrix and an overall SRMR≤.08 . The final samples are described in the Results section. Measurement models were fitted to the homogeneous pooled matrix. When examining the heterogeneity, no pattern could be found that applied equally to all excluded samples (there were no items that were equally larger or smaller in relation to the others). Details regarding divergences and results when all samples are included can be found in the OSF repository.

For TSSEM stage two models, RMSEA≤.06 , SRMR≤.08 indicated a good fit (Hu and Bentler, 1999). Due to the WLS estimator, the interpretation of the CFI should be treated with caution (Cheung, 2015a). A non-significant test statistic indicates a perfect fit. However, large samples tend to produce significant results (Kline, 2023). Therefore, a non-significant test statistic was not expected. The results of the second step were used to investigate the latent structure of the questionnaires. This is done by comparing the Akaike information criterion (AIC) and the Bayesian information criterion (BIC). For both, lower values indicate a better fit. In general, construct validity is considered to exist when different methods of measuring the same characteristic are highly correlated with each other (convergent validity) and when different methods measuring different characteristics are low correlated (discriminant validity). According to Hair (2019), this can be quantified in a measurement model using the average variance extracted (AVE).


$$AVE=\frac{\sum_{i=1}^{n}L_{i}^{2}}{n\text{.}}$$

$L_{i}$ is the completely standardized factor loading of the item i , and n is the number of items measuring a factor. It should be at least 0.50 for each factor to assume convergent validity of the factor. This would mean that, on average, at least 50% of the variance of each items associated with that factor is explained by it. The Fornell-Larcker criterion is used to assess whether a scale factor is discriminant from the other scale factors. It states that discriminant ability exists when the AVE is greater than any squared correlation with another construct (Fornell and Larcker, 1981). In addition, we evaluate the interscale and intrascale MIC based on the pooled correlation matrix. For a broad construct, the MIC of items within the same scale should be greater than 0.15 for convergent validity (Clark and Watson, 2019). Higher intrascale correlations relative to interscale correlations support the separation of constructs. Factor and total test reliability is calculated using a congeneric measurement model and reported as congeneric reliability (Cho, 2016).

All of these calculations were based on the best-fitting model. Supplementary Table 2 shows the congeneric reliability formulas for different measurement models. In addition to the aggregated characteristic values, the coefficients and residual errors were considered. Because of the large pooled sample size, even small estimates are likely to be statistically significant (Kline, 2023). The practical significance is even more important. Standardized factor loadings are said to be meaningful if they are at least |0.30|, adequate if they are at least |0.50|, and good if they are at least |0.70| (Hair, 2019). Values less than |0.10| are considered equal to zero. A value of |0.30| means that the factor explains 9% of the variance of the indicator and that the item reliability is 0.09. Values of |0.50| and |0.70| mean an explanation of 25% and. 49%, respectively.

## 3 Results

### 3.1 Sample characteristics

Table 1 provides details on the final samples included. An overview of all identified studies can be found in the OSF repository. All SD3 samples used an item scale of 1 to 5. Therefore, the possible range is 9 to 45 per scale. One of the samples contains only the narcissism scale. Six SD3 samples have missing entries. A total of 507 values were imputed. The correlations between the Machiavellianism and psychopathy manifest scores are high, across samples (range: 0.47 to 0.68). Between Machiavellianism and narcissism, as well as between psychopathy and narcissism, the range of correlation values is broader (range: 0.23 to 0.45, respectively, 0.18 to 0.53).

**Table 1:** Sample characteristics.

Short dark triad questionnaire^a^									
Reference (Sample)	Country	N	*M_Age_ (SD_Age_)*	*M_M_ (SD_M_)*	*M_N_ (SD_N_)*	*M_P_ (SD_P_)*	Cor_M,N_	Cor_M,P_	Cor_N,P_
Bardeen and Michel (2019) (1)	USA	579	35.60 (11.30)	25.40 (6.32)	23.50 (6.24)	18.60 (6.26)	0.35	0.64	0.41
Bardeen and Michel (2019) (2)	USA	597	35.20 (10.80)	24.20 (6.77)	22.40 (6.12)	18.60 (6.20)	0.45	0.59	0.53
Semenyna et al. (2019)	CA	2,046	20.61 (3.76)	25.50 (4.61)	26.93 (5.03)	17.77 (4.65)	0.24	0.45	0.25
Szabó and Jones (2019)	USA	972	20.67 (4.51)	27.27 (5.42)	26.78 (4.75)	19.38 (5.16)	0.23	0.52	0.18
Armstrong et al. (2020)	USA	866	20.35 (2.78)	26.58 (5.82)	26.95 (4.65)	17.79 (4.61)	0.35	0.49	0.27
Garcia et al. (2020) (2)	USA	2,372	34.13 (11.92)	23.90 (5.55)	24.50 (6.02)	15.40 (5.14)	0.35	0.53	0.40
Hart and Richardson (2020)	USA	567	19.07 (NA)	25.30 (6.33)	26.60 (5.48)	19.10 (5.60)	0.30	0.50	0.27
Hayes et al. (2020)(1)	USA	540	19.27 (1.36)	26.12 (5.89)	25.71 (5.05)	19.45 (5.85)	0.32	0.47	0.34
Miller et al. (2020) (1)	USA	591	37.00 (11.80)	NA	22.70 (6.71)	NA	NA	NA	NA
Zeigler-Hill et al. (2020) (2)	USA	792	19.89 (3.38)	24.07 (6.12)	24.74 (5.72)	17.47 (5.31)	0.36	0.55	0.33
Zeigler-Hill et al. (2020) (3)	USA	755	25.42 (9.24)	24.07 (6.66)	24.55 (5.74)	18.08 (6.69)	0.45	0.68	0.38
Kjeldgaard-Christiansen et al. (2021) (1)	USA, CA	1,805	NA	26.10 (6.94)	22.30 (6.89)	18.00 (6.51)	0.44	0.59	0.47
Pfattheicher et al. (2021) (1c)	USA	985	38.50 (12.40)	24.00 (7.01)	21.80 (6.60)	14.30 (5.21)	0.31	0.54	0.40
Sum		13,467		24.07 (7.89)	24.64 (6.13)	16.67 (6.64)	0.30	0.70	0.34
Dirty dozen questionnaire^b^									
Clancy et al. (2020)	USA, CA	469	22.43 (3.17)	11.94 (5.81)	14.15 (6.23)	10.20 (5.55)	0.57	0.63	0.43
Garcia et al. (2020) (1)	USA	1,000	31.5 (10.27)	13.49 (5.40)	15.30 (5.31)	10.95 (5.13)	0.43	0.49	0.21
Garcia et al. (2020) (2)	USA	309	30.97 (9.63)	13.60 (5.92)	15.74 (5.39)	10.95 (5.30)	0.39	0.55	0.18
Mayor et al. (2020)	USA	326	38.36 (10.49)	11.16 (5.46)	12.90 (5.52)	10.4 (5.38)	0.55	0.61	0.42
Hardin et al. (2021)	USA	411	45.38 (16.29)	10.16 (5.34)	11.08 (5.46)	8.63 (4.80)	0.55	0.60	0.38
Rogoza et al. (2021)	USA	212	19.33 (1.44)	13.25 (5.48)	14.97 (5.13)	10.20 (4.78)	0.49	0.60	0.32
Sum		2,727		10.45 (4.56)	11.82 (4.74)	8.84 (4.16)	0.56	0.60	0.38

M, SD, and Cor with subscripts are mean, standard deviation, and Pearson’s correlation, calculated from the available data. Due to missing data in some datasets, the age was taken from the respective publications. If more than one study is reported in a publication, (sample) refers to the sample of the respective study number. M/N/P = Machiavellianism/Narcissism/Psychopathy.

a Scale range 9–45 for all samples.

b Response range harmonized for all samples, resulting in scale range 4–28.

Different item scales were used for the DD: in one case a scale from 1 to 5 (Mayor et al., 2020), in four cases from 1 to 7 (Garcia et al., 2020; Hardin et al., 2021; Rogoza et al., 2021), and in one case from 1 to 9 (Clancy et al., 2020). For better compatibility, they were harmonized, to a response of 1 to 7, resulting in a possible scale range of 4 to 28. The validity, reliability, and model fit parameters changed minimally when the datasets with a different item scale were excluded (sensitivity analysis in OSF). In addition, it did not lead to any different conclusions. It was therefore decided to include these datasets in the final analyses. In sum, we imputed 13 values. The correlations between the Machiavellianism and psychopathy manifest scores are high across samples (range: 0.49 to 0.63). Again, the correlations between Machiavellianism and narcissism, as well as psychopathy and narcissism are more variable (range: 0.39 to 0.57, respectively, 0.18 to 0.43).

### 3.2 Pooled sample correlations

The first step of TSSEM is to pool the inter-item correlation matrices of all samples. Table 2 shows the summarized information of these matrices. The upper part shows the results of the pooled matrix of the SD3 samples. The complete homogeneous pooled matrices can be found in the OSF repository. The test statistics ( $\chi^{2}=8,439,N=13,467,df=3743,\;p\leq .001;\mathrm{RMSEA}=.035;\mathrm{SRMR}=0.064$ ) confirm that we obtain sufficient homogeneity of the SD3 sample matrices. The summarized pooled matrix of the DD samples is shown in the lower part of Table 2. Again, the test statistics confirm the homogeneity of the inter-item correlations across the sample matrices ( $\chi^{2}=629,N=2,727,df=330,p\leq .001;\mathrm{RMSEA}=.045;\mathrm{SRMR}=0.068$ ). Samples of both questionnaires were selected according to the procedure described in the Data Analysis section. Pooled matrices including the excluded samples are available in the OSF repository.

**Table 2:** Pooled correlation summaries.

Scale	Items	Intra MIC	MIC_N,P_	MIC_M,P_	MIC_M,N_
SD3-M	1–9	0.30* (0.12, 0.65)	–	0.21* (−0.10, 52)	0.13* (−0.08, 0.41)
SD3-N	10–18	0.25* (0.11, 0.44)	0.13* (−0.04, 0.33)	–	–
SD3-P	19–27	0.28* (0.08, 0.52)	–	–	–
DD-M	1–4	0.52* (0.39, 0.68)	–	0.37* (0.21, 0.55)	0.32* (0.27, 0.48)
DD-N	9–12	0.51* (0.36, 0.66)	0.21* (0.08, 0.39)	–	–
DD-P	5–8	0.49* (0.32, 0.67)	–	–	–

Intra = intrascale; MIC = mean inter-item correlation. MICs with subscripts are interscale mean inter-item correlations. M/N/P = Machiavellianism/Narcissism/Psychopathy. Asterisks indicate significant differences from zero ( p≤0.05 ). MICs in bold indicate significant differences between interscale and intrascale MICs ( p≤0.05 ), verified by the differences between the z-transformed averaged correlations and the standard error of this difference. Parentheses contain the range of individual inter-item correlations. Duplicates have been omitted.

### 3.3 Reliability, convergent, and discriminant validity

In the second stage of TSSEM, the assumed measurement models were fitted to the pooled matrices. The upper part of Table 3 shows the goodness-of-fit indices for the models, fitted to the pooled correlation matrix of the SD3 samples. For none of the models, could the exact fit hypothesis be retained. Based on our criteria, no model provided a good fit. The misfit of the model with three correlated first-order factors (model a) indicates that the scales do not measure unidimensional constructs. Based on AIC, BIC, RMSEA, and SRMR, the orthogonal bifactor model (model b) fits better than the model with three correlated first-order factors. None of the bifactor-(S-1) models (model c) provide a better fit. The best-fitting model, according to these indices, is the bifactor-(S·I-1) model (model d). However, it does not meet the criteria for a good fit either.

**Table 3:** Goodness-of-fit indices for models fitted to pooled correlation matrices.

Model	Ref	χ^2^	df	*p*	RMSEA	SRMR	AIC	BIC
SD3-a		12.845	321	≤ 0.001	0.054	0.111	12.203	9.793
SD3-b		8.967	297	≤ 0.001	0.047	0.088	8.373	6.143
SD3-c	M	10.064	305	≤ 0.001	0.049	0.098	9.455	7.165
SD3-c	P	9.856	305	≤ 0.001	0.048	0.094	9.246	6.956
SD3-c	N	11.318	305	≤ 0.001	0.052	0.096	10.708	8.418
SD3-d	I24	8.375	295	≤ 0.001	0.045	0.088	**7.785**	**5.570**
DD-a		1.125	51	≤ 0.001	0.088	0.139	1.023	721
DD-b		525	42	≤ 0.001	0.065	0.071	441	192
DD-c	M	687	45	≤ 0.001	0.072	0.086	597	331
DD-c	P	600	45	≤ 0.001	0.067	0.066	510	244
DD-c	N	1.012	45	≤ 0.001	0.088	0.127	922	656
DD-d	I4	408	40	≤ 0.001	0.058	0.048	**328**	**92**

Ref = Reference. M/N/P = Machiavellianism/Narcissism/Psychopathy. I24 = Item 24. I4 = Item 4. Model a = correlated factor model. Model b = orthogonal bifactor model. Model c = bifactor-(S - 1) model. Model d = bifactor-(S · I - 1) model. RMSEA = Root Mean Square Error of Approximation. SRMR = Standardized Root Mean Squared Residual. AIC = Akaike Information Criterion. BIC = Bayesian Information Criterion. Bold values indicate the lowest AIC and BIC. SD3: *N* = 13,467 DD: *N* = 2,727.

Table 4 summarizes the scale reliability, AVE, and squared factor correlation estimates of the bifactor-(S·I-1) model, which is the best fit of our measurement models. This is true for both SD3 and DD data. The upper part shows the calculations for the SD3. On the one hand, it shows that the convergent validity of the factors is remarkably low. Thus, on average, the scale-specific factors do not explain much of the item variance. Furthermore, the proportion of the scale-specific factors in the reliabilities ( ${\;}_{p}\rho_{BF}$ % Group Factor) is low, while the proportion of the GRF ( ${\;}_{p}\rho_{BF}$ % General Factor) is high. Thus, a large part of the total scale variance is explained by the GRF. It can also be seen that the scale-specific factors share little variance ( $\phi^{2}$ ). Thus, most of the shared variance of the items is found in the GRF, which quantifies the shared variance with item 24. Third, the often quoted minimum reliability of 0.80 for the scales can only be achieved if the variance that comes from both the GRF and its corresponding scale-specific factor is taken into account. The $\omega_{HBF}$ of GRF ${\;}_{I24}$ is 0.84, and $\rho_{BFS-1}$ is 0.92. This also shows that the shared variance with item 24 explains a large proportion of the total item variance. The fourth observation concerns the discriminant validity, as assessed by the Fornell-Larcker criterion. The AVE of the scale-specific factors narcissism and psychopathy is higher than the shared variance between them ( $\phi_{Res_{N},\mathrm{ResP}}^{2}$ ). This is also true for the shared variance between $Res_{M}$ and $Res_{N}$ but not for the AVE and the shared variance of $Res_{M}$ and $Res_{P}$ . It follows that $Res_{N}$ contains variance that distinguishes it from the other scale-specific factors. On the other hand, $Res_{P}$ and $Res_{M}$ share more variance than they explain on average for their own items. The reported reliabilities for the SD3 are only valid for the bifactor-(S·I-1) model.

**Table 4:** Estimators for scale reliability and AVE of the bifactor-(S · I − 1) model.

						${\;}_{p}\rho_{BF}$		
Scale	Items	AVE	$\phi_{Res_{N},Res_{P}}^{2}$	$\phi_{Res_{M},Res_{P}}^{2}$	$\phi_{Res_{M},Res_{N}}^{2}$	Total	GRF	Group factor
SD3-Res ${\;}_{M}$	1–9	0.09	–	0.23	0.06	0.86	0.72	0.14
SD3-Res ${\;}_{N}$	10–18	0.19	0.13	–	–	0.80	0.33	0.47
SD3-Res ${\;}_{P}$	19–23, 25–27	0.07	–	–	–	0.80	0.71	0.09
SD3-GRF ${\;}_{I24}$	1–27	0.28	–	–	–	–	–	–
DD-Res ${\;}_{M}$	1–3	0.18	–	0.05	0.09	0.82	0.62	0.20
DD-Res ${\;}_{N}$	9–12	0.39	0.04	–	–	0.85	0.30	0.54
DD-Res ${\;}_{P}$	5–8	0.19	–	–	–	0.83	0.58	0.25
DD-GRF ${\;}_{I4}$	1–12	0.37	–	–	–	–	–	–

AVE, average variance extracted by scale-specific factor of the variables. $\phi^{2}$ with subscript is shared variance between scale-specific factors. SD3-Res = Short Dark Triad scale-specific factor. DD-Res, Dirty Dozen scale-specific factor. GRF = general reference factor. M/N/P = Machiavellianism/narcissism/psychopathy. ${\;}_{p}\rho_{BF}$ = congeneric scale reliability. Duplicates have been omitted. ${\;}_{p}\rho_{BF}$ is not reported for GRF, appropriate are $\omega_{HBF}$ and $\rho_{BFS-1}$ , which are reported in text.

The lower part of Table 3 shows the goodness-of-fit indices for the measurement models, fitted to the pooled correlation matrix of the DD samples. As expected, none of the models pass the exact fit hypothesis. According to the global fit indices, the orthogonal bifactor model (model b) fits better than the correlated factor model (model a). The bifactor-(S-1) models (model c) with Machiavellianism and narcissism as references have a worse fit. The bifactor-(S-1) model with psychopathy as reference has a similar fit to the orthogonal bifactor model, with a slightly lower SRMR value. However, AIC and BIC favor model b. The best fit is the bifactor-(S·I-1) model (model d), with item 4 as the reference. According to the AIC and BIC, this model fits the data best and should be selected. In addition, model d was the only model that met our criteria for a good fit.

At the bottom of Table 4 are the aggregated estimates of scale reliability, AVE, and squared factor correlation for the DD data. It shows the following points. First, the convergent validity of the factors is remarkably low. Consequently, on average, the scale-specific factors have limited explanatory power over the item variance. Second, the proportion of variance accounted for by the scale-specific factors ( ${\;}_{p}\rho BF$ % Group Factor) is comparatively small for Machiavellianism and psychopathy and comparatively large for narcissism, whereas the GRF accounts for a significant proportion ( ${\;}_{p}\rho_{BF}$ % General Factor) for all scales. Thus, the GRF contributes significantly to the total variance of the scales. In addition, the scale-specific factors share little variance ( $\phi^{2}$ ). Thus, the majority of the shared variance among all items can be attributed to the GRF, which quantifies the shared variance with item 4. Third, the often quoted minimum reliability of 0.80 for the scales can only be achieved if the variance that comes from both the GRF and its corresponding scale-specific factor is taken into account. The ωHBF of GRF ${\;}_{I}4$ is 0.79, while $\rho_{BFS-1}$ is 0.93. This observation further indicates that a significant portion of the total item variance is explained by the shared variance with item 4. Furthermore, the Fornell-Larcker criterion is met for all scale-specific factors of the DD. Accordingly, they can be seen as distinct from each other. It should be noted that the reliabilities for the DD are only valid when assuming this model, which has an acceptable global fit to our data.

### 3.4 SD3 coefficients

In the following, the three best-fitting models are compared based on their factor loadings (for the others, see OSF repository). These are the three different bifactor models: orthogonal, (S-1), and the (S·I-1). Table 5 shows the standardized coefficients for the SD3 models. The reference factor for the ( S−1 ) model is psychopathy, and the reference item for the (S·I-1) model is item 24, from the psychopathy scale.

**Table 5:** Standardized parameter estimates for different bifactor models for SD3 data.

I /	Orthogonal				Bifactor-(S-1)			Bifactor-(S·I-1)			
F	M	N	P	DT	M_*Res*_	N_*Res*_	GRF_*Psych*_	M_*Res*_	N_*Res*_	P_*Res*_	GRF_*I*24_
01	0.42*	–	–	0.18*^a^	0.40*	–	0.17*	0.44*	–	–	0.20*
02	0.11*^a^	–	–	0.77*	0.12*,^a^	–	0.76*	0.03*^a^	–	–	0.77*
03	0.15*^a^	–	–	0.68*	0.13*,^a^	–	0.68*	0.10*^a^	–	–	0.69*
04	0.32*	–	–	0.43*	0.31*	–	0.42*	0.32*	–	–	0.44*
05	0.27*	–	–	0.77*	0.31*	–	0.76*	0.27*^a^	–	–	0.77*
06	0.23*^a^	–	–	0.78*	0.27*,^a^	–	0.76*	0.27*^a^	–	–	0.77*
07	0.53*	–	–	0.36*	0.52*	–	0.35*	0.51*	–	–	0.38*
08	0.18*^a^	–	–	0.52*	0.17*,^a^	–	0.52*	0.16*^a^	–	–	0.53*
09	0.22*^a^	–	–	0.53*	0.23*,^a^	–	0.53*	0.17*^a^	–	–	0.54*
10	–	0.48*	–	0.27*^a^	–	0.48*	0.27*	–	0.48*	–	0.27*
11	–	0.59*	–	0.17*^a^	–	0.58*	0.20*	–	0.62*	–	0.17*
12	–	0.39*	–	0.53*	–	0.39*	0.53*	–	0.41*	–	0.52*
13	–	0.42*	–	0.49*	–	0.43*	0.49*	–	0.42*	–	0.49*
14	–	0.37*	–	0.50*	–	0.35*	0.50*	–	0.31*	–	0.51*
15	–	0.52*	–	0.04*^a^	–	0.52*	0.05*	–	0.53*	–	0.04*
16	–	0.29*^a^	–	0.46*	–	0.29*^a^	0.46*	–	0.31*	–	0.45*
17	–	0.40*	–	0.22*^a^	–	0.41*	0.27*	–	0.45*	–	0.22*
18	–	0.27*^a^	–	0.47*	–	0.26*^a^	0.46*	–	0.21*^a^	–	0.48*
19	–	–	0.23*^a^	0.69*	–	–	0.73*	–	–	0.11*	0.71*
20	–	–	0.36*	0.16*^a^	–	–	0.25*^a^	–	–	0.53*	0.18*^a^
21	–	–	0.16*^a^	0.73*	–	–	0.75*	–	–	−0.04*	0.74*
22	–	–	0.36*	0.56*	–	–	0.63*	–	–	0.24*	0.59*
23	–	–	0.16*^a^	0.55*	–	–	0.58*	–	–	**−0.01**	0.57*
24	–	–	0.08*^a^	0.76*	–	–	0.76*	–	–	–	0.76*
25	–	–	0.38*	0.12*^a^	–	–	0.20*^a^	–	–	0.33*	0.16*^a^
26	–	–	0.33*	0.44*	–	–	0.50*	–	–	0.24*	0.47*
27	–	–	0.13*^a^	0.73*	–	–	0.75*	–	–	0.06*	0.74*
M ${\;}_{Res}$	–	–	–	–	–	−0.10*	–	–	−0.25*	−0.48*	–
N ${\;}_{Res}$	–	–	–	–	–	–	–	–	–	0.36*	–
P ${\;}_{Res}$	–	–	–	–	–	–	–	–	–	–	–

The variances of the latent variables were fixed at 1, correlation matrices were used as input, so the loadings correspond to the fully standardized solution. Asterisks indicate statistically significant differences from zero (*p* ≤ 0.05). Bold values indicate no practical significance (λ < 0.30). I/F = Item/Factor. Subscripts M/N/P = Machiavellianism/narcissism/psychopathy. GRF = general reference factor. M_Res_/N_Res_/P_Res_, respective scale-specific factor. Estimates between M/N/P_Res_ represent correlations. Duplicates have been omitted.

a Notable regarding model assumptions, see section SD3 Coefficients for details.

In the orthogonal bifactor model, all factor loadings are statistically significant, but not all are meaningful ( $\lambda_{i}\geq |.30|$ ). Some items are explained to a substantial degree by both the specific and general factors. Many, however, are explained by only one or the other. We have two coefficients close to zero ( $\lambda_{15DT},\lambda_{24P}$ ).

Most items of the Machiavellianism scale are well explained by the general DT factor. Only item 01 is not. The specific Machiavellianism factor explains only a relevant part of the variance in items 01, 04, and 07. Items 04 and 07 have substantial loadings on both general and specific factors. In the narcissism scale, items 10, 11, 15, and 17 are well explained by the specific factor but not by the general factor. The coefficient of the general DT factor on item 15 is close to zero. Items 16 and 18 are well explained by the general factor but not by the specific factor. Items 12, 13, and 14 are well explained by both factors. Regarding the psychopathy scale, the items 20 and 25 are only explained to a relevant extent by the specific factor. Items 19, 21, 23, 24, and 27 are mainly explained by the general DT factor. The specific coefficient of item 24 is close to zero. Items 22 and 26 are well explained by both factors.

A similar picture emerges for the bifactor-(S-1) model. In the form that the coefficients of the DT are almost identically mapped onto the reference factor of psychopathy. Regarding the Machiavellianism scale, almost all coefficients are of the same magnitude as in the orthogonal bifactor model. One difference is item 05, where the coefficient on the residual scale factor is now also practically significant. However, the difference is minimal. Some items of the psychopathy scale (item 20 and item 25) are not well explained by the general reference factor. Most of the psychopathy scale items that show a clear pattern in the orthogonal bifactor model with respect to the general factor show almost no change with respect to the reference factor. For narcissism, there are no noteworthy differences. Items whose variance in the orthogonal bifactor model was explained exclusively or in addition to DT by the specific factor show clearer changes, however, not enough to affect the interpretation of their significance. The correlation between the latent residual scale factors is negative but negligibly small.

In the bifactor-(S·I-1) model, we quantify the variance shared with a reference item. We chose item 24, which belongs to the psychopathy scale, because in the orthogonal bifactor model the coefficient of the specific factor was close to 0, while the loading on the general factor was very high. Again, the coefficients of the scale-specific factors are very similar to the specific coefficients in the orthogonal bifactor model. The specific coefficient of item 16 is now greater than the cutoff we defined as meaningful, but it is a marginal increase.

More interesting are the non-reference psychopathy items. Three items (21, 23, and 27) have scale-specific factor loadings close to zero. Items 22 and 26, which were explained by both factors in the orthogonal bifactor model, are now explained at substantial levels only by the general reference factor. But again, it is only a marginal increase. There is a small but notable change in item 20. It shows a higher coefficient on the scale-specific factor than on the specific factor in the orthogonal bifactor model (0.53 vs. 0.36). The correlations between the Machiavellianism and psychopathy scale-specific factors are negative and of moderate strength. There is a small negative relationship between the scale-specific factors of Machiavellianism and narcissism. The scale-specific factors of psychopathy and narcissism are moderately positively related.

### 3.5 DD coefficients

Table 6 shows the factor loadings for the three best-fitting DD measurement models: the orthogonal bifactor model, the bifactor-(S-1) model, with psychopathy as the reference, and bifactor-(S·I-1) model, with item 4 as the reference.

**Table 6:** Standardized parameter estimates for different bifactor models for DD data.

I /	Orthogonal				Bifactor-(S-1)			Bifactor-(S·I-1)			
F	M	N	P	DT	M_*Res*_	N_*Res*_	GRF_*Psych*_	M_*Res*_	N_*Res*_	P_*Res*_	GRF_*I*4_
01	0.14*^a^	–	–	0.80*	0.52*	–	0.66*	0.15*	–	–	0.81*
02	0.56*	–	–	0.67*	0.54*	–	0.54*	0.33*	–	–	0.67*
03	0.33*	–	–	0.54*	0.59*	–	0.34*	0.64*	–	–	0.48*
04	−0.06*^a^	–	–	0.89*	0.39*	–	0.75*	–	–	–	0.87*
05	–	–	0.56*	0.63*	–	–	0.84*	–	–	0.56*	0.65*
06	–	–	0.51*	0.60*	–	–	0.80*	–	–	0.49*	0.62*
07	–	–	0.45*	0.65*	–	–	0.80*	–	–	0.40*	0.68*
08	–	–	0.26*^a^	0.47*	–	–	0.54*	–	–	0.17*^a^	0.50*
09	–	0.74*	–	0.38*	–	0.81*	0.19*	–	0.77*	–	0.34*
10	–	0.71*	–	0.36*	–	0.78*	0.19*	–	0.73*	–	0.33*
11	–	0.58*	–	0.48*	–	0.67*	0.34*	–	0.59*	–	0.46*
12	–	0.28*^a^	–	0.66*	–	0.42*	0.58*	–	0.28*^a^	–	0.66*
M ${\;}_{Res}$	–	–	–	–	–	0.52*	–	–	0.31*	−0.22*	–
N ${\;}_{Res}$	–	–	–	–	–	–	–	–	–	−0.20*	–
P ${\;}_{Res}$	–	–	–	–	–	–	–	–	–	–	–

The variances of the latent variables were fixed at 1, correlation matrices were used as input, so the loadings correspond to the fully standardized solution. Asterisks indicate statistically significant differences from zero (*p* ≤ 0.05). Bold values indicate no practical significance (λ < 0.30). I/F = Item/Factor. Subscripts M/N/P, Machiavellianism/narcissism/psychopathy. GRF = general reference factor. M/N/P_Res_ = respective scale-specific factor. Estimates between M/N/P_Res_ represent correlations. Duplicates have been omitted.

a Notable regarding model assumptions, see section DD Coefficients for details.

In the orthogonal bifactor model, the specific factor Machiavellianism shows an unexpected loading structure that is not in line with theoretical assumptions (negative and near-zero loadings). At the same time, we have statistically significant loadings of considerable magnitude on the common DT factor. For the psychopathy items, each loading is statistically significant. The specific loading of item 08 is just below the defined threshold of practical significance. The narcissism items are well explained by both factors.

In the bifactor-(S-1) model, most loadings are significant and at a substantial level. Only items 09 and 10 (narcissism) are not well explained by the psychopathy reference factor. The scale-specific factors for Machiavellianism and narcissism are highly correlated. The Machiavellianism items do not show the unexpected pattern of the orthogonal bifactor model.

For the bifactor-(S·I-1), item 04 (Machiavellianism scale) was chosen as the reference. The Machiavellianism items do not show the unexpected pattern of the orthogonal bifactor model. The model shows that much of the variance of the Machiavellianism and psychopathy items is shared with item 04. In addition, items 01 and 08 do not share any relevant amount of variance with the other items in their respective scales. The others do. Of the narcissism scale, only the variance of item 12 is mainly explained by the GRF. The other items have common variance of relevant amount, which is about the common variance with item 04.

## 4 Discussion

In the present study, we examined two prominent short scales for measuring the DT of personality. Using a meta-analytic approach, we examined several measurement models to determine whether they adequately represent the data. We then examined the psychometric properties of the instruments at the item level. In the following sections, we consider the implications of our findings for our research question and relate them to other studies.

### 4.1 Psychometric properties of the SD3

The pooled correlation matrices provide insight into the properties of both instruments at the manifest level across multiple samples. In the current data, the intrascale MIC meets the threshold for convergent validity, but individual item-to-item correlations within the same scale reveal that some items are weakly associated with others. This suggests limited homogeneity within certain dimensions, which aligns with the observed misfit of a model with three correlated factors. The interscale MICs are lower than the intrascale MICs, which indicates that, on average, the scales are reasonably well-separated. This supports the notion of a multidimensional construct with three distinct factors.

However, a closer inspection of individual item-to-item correlations reveals notable overlap at the item level, despite the aggregated MIC metrics suggesting distinct factors. Specifically, several Machiavellianism items exhibit strong cross-correlations with items from other scales. For example, items 02, 05, and 06 show cross-correlations greater between 0.40 and 0.52 with items 19, 21, 24, and 27, while item 03 shows a correlation of 0.41 with item 14. These cross-correlations suggest that these items may align more closely with the content of other scales, challenging their assignment to their current scale.

At the latent level, discriminant validity is evaluated through global model fit and the relationship between the AVE and interfactor correlations (Fornell-Larcker criterion; Fornell and Larcker, 1981; Hair, 2019). In this study, the misfit of the measurement model with three correlated factors does not support the discriminability or the multidimensionality of the items with respect to this three-factor structure. Moreover, the extracted variance of the factors is largely shared among them ( $\phi_{MP}^{2}=0.85$ , $\phi_{MN}^{2}=0.52$ , $\phi_{NP}^{2}=0.53$ , see OSF for more details). This is consistent with previous findings of Miller et al. (2017), who reported that Machiavellianism and psychopathy share 80% of their variance in a similar three-factor model.

SD3 narcissism, on the other hand, appears to be quite distinct from the other scales. In all reported models, there is a correlation with the other two constructs, as hypothesized in the theory (Paulhus and Williams, 2002). In the bifactor-(S·I-1) model, these items are also explained to a relevant extent by their own scale-specific factor. It is worth noting that some items that share variance with the reference item are also explained to a significant degree by the scale-specific residual factor and that there are additional items that are explained only by this factor. This does not contradict the theory, but it does show that the unidimensional conception of SD3 narcissism also falls short.

Regarding the reliability coefficients, the bifactor-(S·I-1) model gives values that are generally considered to be good. This is true for all scales and the overall model. Most of the total item variance is explained by the GRF based on item 24. For both the Machiavellianism and the psychopathy scales, this factor accounts for more than 70% of the variance, while the respective scale-specific factors each contribute less than 15%. This leads to the conclusion that, after partializing out the shared variance with item 24, there is a comparatively small but substantial amount of shared variance in either the remaining psychopathy items or the Machiavellianism items. The frequently postulated recommendation to combine these two scales into a single factor (Persson et al., 2019; Sharpe et al., 2021) is not supported by our findings. Although psychopathy and Machiavellianism are closely related, some high specific factor loadings and negative correlations between specific factors argue against this. SD3 Narcissism is well discriminated. Although there is common variance with item 24, a substantial proportion (almost 50%) shares items in this scale but not with item 24.

Although the model shows a good approximate fit according to the RMSEA, the other fit criteria show that the model does not fit the data very well. The results of this model should be treated with caution. The misfit of all models considered shows that the items of the SD3 do not follow the intended theoretical structure and that this questionnaire may not be suitable for measuring the DT. Specifically, it is about the items 02, 03, 05, 06, 08, and 09, which—although assigned to the Machiavellianism scale—are more related to the psychopathy items (S-1 model), or to item 24 of the psychopathy scale (S·I-1 model). Same is true for item 18 of the narcissism scale. Our findings indicate that items that are directed toward revenge (e.g., item 24 “People who mess with me always regret it.,” Jones and Paulhus, 2014) or influencing others (e.g., item 5 “It’s wise to keep track of information that you can use against people later.” Jones and Paulhus, 2014) lead to the empirical overlap between Machiavellianism and psychopathy.

### 4.2 Psychometric properties of the DD

The pooled correlation matrix of the DD shows good construct validity. All items of a scale are highly correlated with each other, while the MIC with items of other scales is lower. However, the assumption of three correlated homogeneous dimensions must be rejected due to the misfit of the model with three correlated factors.

The interscale MICs indicate that the scales are discriminant. This is because they are lower than the intrascale MICs. This is consistent with the idea of a multidimensional construct with three factors. However, the individual interscale correlations between Machiavellianism and psychopathy and between Machiavellianism and narcissism are also consistently in the high range. Thus, the Machiavellianism items are more highly correlated with each other than with items outside the respective scales, but they are also highly correlated with the other scales. The cross-correlations ultimately lead to the misfit of a model with three correlated factors.

In the bifactor-(S·I-1) model, we see that the scale-specific factors explain slightly more item variance than in the SD3. The explained variance of the Machiavellianism and psychopathy factor is in the low double digits, and the Narcissism residual factor explains on average almost 40% of the item variance. The shared variance between the factors is in the single-digit percentage range. According to the Fornell-Larcker criterion, this suggests that these factors are discriminant.

The bifactor-(S·I-1) also gives a high reliability for each scale and also for the overall model. The reference factor accounts for a large proportion of the total test variance and of the scale-specific factors for Machiavellianism and psychopathy. For both Machiavellianism and psychopathy, approximately 60% of the variance is explained by the reference factor, while approximately 20 and 25%, respectively, are explained by the scale-specific factors. In the case of narcissism, more than 50% is explained by the specific factor and 30% by the GRF. It can thus be seen that in DD, too, a large part of the variance in psychopathy and Machiavellianism can be explained by a general reference factor. In general, the bifactor-(S·I-1) model approximately fits the items of this scale. Therefore, the DD seems to be suitable for measuring the DT.

### 4.3 Latent structure of DT questionnaires

The second research question follows the existing discourse whether there is a general factor of the DT (e.g., Moshagen et al., 2018; Vize et al., 2021). This study addresses the question of whether the two most prominent instruments are capable of capturing such a structure. We also discuss methodological and conceptual aspects of estimating a general factor.

Often, an orthogonal bifactor model is fitted to DT data (e.g., Jonason and Luévano, 2013; McLarnon and Tarraf, 2017; Vaughan et al., 2019). It fits intuitively with idea that if the common part (quantified as general factor) is partialized out, the specific factors are unrelated (i.e., uncorrelated). Most studies conclude that an orthogonal bifactor model best reproduces the SD3 and DD data and justify this on the basis of global fit (Jonason and Luévano, 2013; McLarnon and Tarraf, 2017; Vaughan et al., 2019). In our case, the orthogonal bifactor model also shows a better fit than the correlated factor model. This holds for both questionnaires. However, introductory literature on measurement models warns that just because you have found a suitable model does not mean that it is the actual (data-generating) model (e.g., Kline, 2023).

A look at the underlying correlation matrices can provide more insight into how the structure comes about. Both instruments show several high cross-correlations between items of different scales. The additional general factor picks up these covariances. The poor fit of the model with three correlated factors confirms that there are no distinct factors but rather a heterogeneous correlation structure.

However, it is not clear what the general factor means in terms of content. This could indeed be a common trait. However, it could also represent irregular inter-item correlations that are in contrast to a construct consisting of distinct traits. Based on the critique of the orthogonal bifactor model (Bonifay and Cai, 2017; Eid et al., 2017), it is very likely that the general factor is a data-driven reference factor defined by one of the specific factors and single indicators. Based on our results, this is confirmed by the following. First, both instruments have at least one specific factor loading close to zero. At the same time, the overall factor loading of the same indicator is very high. This means that after removing a large part of the item variance, there is not much left that could be explained by a specific factor. Second, there are high similarities in the coefficients between different bifactor models. Bifactor models with a GRF specified on the results of the orthogonal model show nearly identical parameter estimates. However, the definition and meaning of the general factors vary considerably.

In SD3, we based our model specification of the bifactor-(S·I-1) models on the data-driven results of the orthogonal bifactor model. This logically leads to almost the same results. In the bifactor-(S-1) model presented, the same factor was defined as the reference to which the reference item in the bifactor-(S·I-1) belongs. If we compare the parameter estimates with a bifactor-(S-1) model, with narcissism as the reference (see OSF repository), we see clearly different coefficients. This also suggests that the general factor in the orthogonal bifactor model is defined by a specific factor, in this case psychopathy. Finally, we must conclude that none of the measurement models tested represent the SD3 data well. This finding indicates significant challenges in interpreting the SD3 scores. This contrasts with the DD, which showed a comparatively better fit and interpretability.

In the orthogonal bifactor model for the DD data, after recoding the initial values, we have one specific loading that is negative and close to zero and three coefficients of the same factor that are positive. It is theoretically unexpected that the loadings of different items belonging to the same factor differ in the sign of their loading. The loading close to zero suggests that the orthogonal bifactor model is a data-driven bifactor-(S·I-1) model, with item 4 (Machiavellianism) as the reference. Accordingly, the bifactor-(S·I-1) model with item 4 as the reference has nearly the same coefficients as the specified orthogonal bifactor model with respect to the general reference factor. The difference is that we no longer have unexpected loading patterns.

Of the models tested, the bifactor-(S·I-1) model can best explain the data structure of both instruments. However, it does not fit for the SD3 data very well. For the DD, the bifactor-(S·I-1) model provides a good global fit. This is consistent with other results showing that item 4 represents the entire scale well (Kajonius et al., 2016). Based on this result and the high reliability ( $\omega_{HBF}$ ) of the GRF ${\;}_{I4}$ , the total test score can be interpreted as a good estimate of what item 4 measures. Based on the wording ‘I tend to exploit others for my own ends’ (Jonason and Webster, 2010), it could be the willingness to exploit others. The model with three correlated factors does not fit well, and the measured scale-specific factors of the bifactor-(S·I-1) model can only be estimated by removing the shared variance with item 4.

### 4.4 Strengths and limitations

This study has several strengths. First, the inclusion of several independent large samples with a similar correlation structure. This allows more general statements to be made about the instruments. Random findings are less likely. Second, we evaluated the psychometric properties at the manifest level, based on items, and at the latent level, based on factors. This allows us to better understand the distribution of variance and assign clear meaning to the factors. Individual indicators that contradict the specified structures can be identified. Another strength is the confirmatory approach. We do not explore new structures. Our priority was not to find a specific model to fit the data. This allowed us to evaluate putative models based on rigorous criteria.

There are, of course, limitations to our approach. First, the selection of the final samples. For stage 2 of a fixed effects TSSEM, it is necessary to have a homogeneous correlation matrix. Otherwise, the parameters of the fitted models would be incorrectly estimated. In our case, there were some studies in which more or less many inter-item correlations were overestimated or underestimated (compared to the majority of studies). We had to exclude them from further analysis to make valid and meaningful inferences from the aggregated data. As a result, there is a lack of generalizability. A random effect TSSEM would be useful to shed light on the reasons for the different correlation matrices. This could be used to identify sample characteristics that have an influence. In one attempt, such a model did not converge. This was probably due to the small number of samples and the large number of parameters. This leads to another limitation: the small number of samples due to the short period in which the studies analyzed were published, the very strict criteria and the lack of response from the authors contacted. However, the overall sample sizes of 13,467 and 2,727 and the low standard errors of the parameters indicate that accurate statements about the measurement structure and psychometric properties are possible. In addition, the first stage of our meta-analysis showed that the included studies were homogeneous and comparable.

In addition, the confirmatory approach limits our ability to explore improvements. Given that our results are based on a limited number of studies, it should also be noted that it is possible that our results underestimate the psychometric problems of the instruments. For example, the range of inter-item correlations is even larger when all samples are considered. This is precisely what led to the exclusion of the outlying samples.

### 4.5 Implications

Our findings and the named limitations have implications for the study of and the work with the DT. Further research should substantiate our findings. For example, it should be checked whether the pattern found in this study generalizes to other samples. It should be noted that potential moderator variables at the study level are preserved in the data. Furthermore, the pooled data could be used for exploratory analysis to see whether an adequate solution for modeling the data can be found. With regard to our first research question, the lack of adequate fit of traditional measurement models raises concerns about their suitability for accurate and reliable measurement of the DT. This suggests the need for caution when using these instruments.

The limitations of the existing instruments and the lack of clarity about the content meaning of the SD3 factors challenge the interpretation of scale and total scores. Because no tested measurement model fits, caution is warranted in making content inferences. Regarding DD, instead of using sum scores, the calculation of factor scores based on factor analysis is recommended for a more appropriate interpretation. Based on the bifactor-(S·I-1) model, a total score would be what item 4 measures (willingness to exploit), the scale scores would be a mixture of what remains (not willingness to exploit). Sum scores can be misleading if the questionnaire does not have a simple structure (DiStefano et al., 2009). A more detailed comparison between sum scores and factor scores is beyond the scope of this study. For further details on this topic, see McNeish and Wolf (2020). The total sum score should not be misinterpreted as representing a common core, nor should the scale sum score be interpreted as representing a specific trait. The meaning of the sum scores is unclear.

In relation to our second research question, our results suggest that a bifactor-(S·I-1) model, with item 4 as reference, is appropriate for DD. Meanwhile, we were unable to find a suitable measurement structure for the SD3. The DD is therefore the preferred measuring instrument. Furthermore, we can conclude that the traditional orthogonal bifactor model is not suitable for evaluating DT personality data, as it fails to adequately represent the underlying structure of both questionnaires. Alternative models should be considered.

In addition to a clear measurement structure, other psychometric criteria may play a role in the selection of the questionnaire. These include predictive and incremental validity. Lee et al. (2012) show predictive ability for both instrument, in terms of short-term mating, power, and money striving. Maples et al. (2014) report that the SD3 explains more variance in the single-construct questionnaires. This can be interpreted as incremental validity and speaks in favor of the SD3. This goes hand in hand with other studies that claim that the DD lacks convergent validity in relation to psychopathy (Miller et al., 2012). The findings of item response theory with regard to discrimination between individuals can also be considered. For both questionnaires, the items primarily differentiate between people with an average level of the latent traits (Persson et al., 2017; Webster and Jonason, 2013).

The results show that existing short questionnaires suffer from the fact that the constructs to be measured are not clearly defined. The lack of a clear and consistent conceptualization of the DT traits hinders progress in understanding them. Recently, several authors have raised this issue. For example, Bader et al. (2022) noted cross-construct overlap between dimensions of single-construct instruments. This suggests that it is not just a problem of constructing the short scales. Miller et al. (2019) criticized the lack of instruments for Machiavellianism, which is actually conceived differently from psychopathy. New single-construct questionnaires have already been developed in response to this issue, such as the Five-Factor Machiavellian Inventory (Collison et al., 2018). Kowalski et al. (2021) explain that the lack of distinction is already a fundamental problem in the conceptualization of dark traits. As described at the outset, this has not limited the use of the instruments examined here. Experts should work toward defining the unique characteristics of each trait and distinguishing them from one another. The development of new instruments that address the challenges of cross-construct overlap and specifically target traits such as Machiavellianism is recommended.

The first step in developing a better instrument would therefore be to define the constructs clearly and unambiguously. As part of this, the question of whether all members of the triad share a common latent core should be answered *a priori*. Currently, the common core is given meaning based on the inappropriate fit of an orthogonal bifactor model to a construct defined as triadic. It would be appropriate to first define this core theoretically to construct a questionnaire that can adequately measure it.

Once these questions have been answered, an instrument with a clear measurement structure and selective items can be constructed. Items should therefore be formulated in such a way that they are highly correlated within their scales and poorly correlated with items in other scales. If the instrument is based on a measurement model that includes a GRF, then the trait-specific factors must be clearly distinct from each other. This could be quantified, for example, using the Fornell-Larcker criterion (Fornell and Larcker, 1981). This ensures that the items in a scale have more in common with each other than the scales have with each other.

Regarding the measurement structure, it should be noted again that orthogonal bifactor models are only appropriate under certain conditions. Eid et al. (2017) have explained in detail why the use of such a model is not appropriate in many psychological domains. Among other things, they point out that a two-stage sampling process is necessary to correctly define a G-factor. However, the usual procedure in DT research is a one-level sampling process: The individuals in the samples are randomly drawn from a set of possible individuals—the constructs and items to be measured, on the other hand, are fixed and are not drawn from a set of possible ones.

Thus, if the self-report form is desired, new short instruments should be explicitly constructed with an appropriate measurement model. Classically, this would be the three correlated factor model. However, if one wishes to retain the idea that the members of the DT share a common latent core, then future instruments should also be constructed in this way. This could be done, for example, by defining a common core a priori, such as low agreeableness. The questionnaire would then have to include items measuring agreeableness. These would then form the GRF in a bifactor-(S-1) model. The trait-specific factors would then contain only the variance not shared with agreeableness.

## 5 Conclusion

The popular DT short instruments (DD, SD3) have reliability and validity problems. The DD fits a bifactor-(S·I-1) model, while the SD3 fails to achieve an adequate measurement model, indicating a need for structural revision. Correlated factor models and orthogonal bifactor models do not fully reflect DT data structures. The orthogonal bifactor model is inappropriate for DT data. It assumes interchangeable traits, and our results confirm that this is not the case. Existing DT short instruments require revision to improve psychometric properties and theoretical alignment. The bifactor-(S·I-1) model provides a viable framework that emphasizes the need for clear definitions of common and specific components. Our study demonstrates for the first time that the orthogonal bifactor model fitted to DT data, from DD and SD3 often reflects a bifactor-(S·I-1) structure, which complicates its interpretation. It follows that it should be avoided because of its restrictive assumptions. The results, based on a relatively small but homogeneous sample of studies with a large number of individual cases, provide solid evidence. However, caution should be exercised in generalizing across contexts.

## Data Availability

Publicly available datasets were analyzed in this study. This data can be found at: https://osf.io/26f39/.

## Footnotes

^1^ https://osf.io/26f39/
