# Supplementary material for: Measuring the dark triad: a meta-analytical SEM study of two prominent short scales
Source: Front Psychol. 2025 Jan 15;15:1469970. doi: 10.3389/fpsyg.2024.1469970 (PMC11774945; doi:10.3389/fpsyg.2024.1469970)
Supplement: Supplementary file 1 [file Table_1.docx]

Supplementary Table 1. Significant Correlations of Dark Triad Traits with Other Personality Traits.

| Dark Triad Trait | Positive Correlate | Negative Correlate | Reference |
| --- | --- | --- | --- |
| NPI Narc | Extraversion  (.33 to .34) | Agreeableness (‑.25 to ‑.34) |  |
|  | Openness (.17 to .20) |  | Muris et al. (2017); Schreiber and Marcus (2020); Vize et al. (2018) |
|  |  | Neuroticism  (‑.12) | Schreiber and Marcus (2020) |
|  |  | Honesty-Humility (‑.59) | Muris et al. (2017) |
| DD Narc | Extraversion  (.14 to .18) |  | Schreiber and Marcus (2020); Vize et al. (2018) |
|  | Neuroticism  (.11) |  | Schreiber and Marcus (2020) |
|  |  | Agreeableness (‑.20 to ‑.16) | Muris et al. (2017); Schreiber and Marcus (2020); Vize et al. (2018) |
|  |  | Honesty-Humility (‑.45) | Muris et al. (2017) |
| SD3 Narc | Extraversion (.48 to .56) |  | Muris et al. (2017); Schreiber and Marcus (2020); Vize et al. (2018) |
|  | Openness  (.18) |  |  |
|  |  | Agreeableness (‑.13 to ‑.10) | Schreiber and Marcus (2020); Vize et al. (2018) |
|  |  | Neuroticism (‑.15 to ‑.13) | Muris et al. (2017); Schreiber and Marcus (2020) |
|  |  | Honesty-Humility (‑.35) | Muris et al. (2017) |
| MACH IV Mach | Neuroticism  (.14 to .17) |  | Muris et al. (2017); Schreiber and Marcus (2020) |
|  |  | Agreeableness  (-.51 to ‑.45) |  |
|  |  | Conscientiousness  (-.27 to -.23) | Muris et al. (2017); Schreiber and Marcus (2020); Vize et al. (2018) |
|  |  | Extraversion  (-.11) | Schreiber and Marcus (2020); Vize et al. (2018) |
|  |  | Honesty-Humility  (-.65) | Muris et al. (2017) |
| DD Mach | Neuroticism  (.10) |  | Schreiber and Marcus (2020) |
|  |  | Agreeableness  (-.37 to ‑.34) |  |
|  |  | Conscientiousness  (-.28 to -.20) | Muris et al. (2017); Schreiber and Marcus (2020); Vize et al. (2018) |
|  |  | Honesty-Humility  (-.60) | Muris et al. (2017) |
| SD3 Mach | Neuroticism  (.13) |  | Schreiber and Marcus (2020) |
|  |  | Agreeableness  (-.44 to ‑.30) | Muris et al. (2017); Schreiber and Marcus (2020); Vize et al. (2018) |
|  |  | Conscientiousness  (-.14 to -.12) | Schreiber and Marcus (2020); Vize et al. (2018) |
|  |  | Extraversion  (-.12) | Vize et al., 2018 |
|  |  | Neuroticism  (-.15) |  |
|  |  | Honesty-Humility  (-.60) | Muris et al. (2017) |
| SRP Psych |  | Agreeableness  (‑.49 to ‑.43) |  |
|  |  | Conscientiousness  (‑.29 to ‑.25) | Muris et al. (2017); Schreiber and Marcus (2020); Vize et al. (2018) |
|  |  | Honesty-Humility  (‑.91) | Muris et al. (2017) |
| DD Psych |  | Agreeableness  (‑.46 to ‑.42) |  |
|  |  | Conscientiousness  (‑.23 to ‑.21) | Muris et al. (2017); Schreiber and Marcus (2020); Vize et al. (2018) |
|  |  | Neuroticism  (-.13) |  |
|  |  | Honesty-Humility  (‑.39) | Muris et al. (2017) |
| SD3 Psych | Neuroticism  (.13) |  | Schreiber and Marcus (2020) |
|  |  | Agreeableness  (‑.49 to ‑.39) |  |
|  |  | Conscientiousness  (‑.26 to ‑.25) | Muris et al. (2017); Schreiber and Marcus (2020); Vize et al. (2018) |
|  |  | Neuroticism  (-.30) |  |
|  |  | Honesty-Humility  (‑.56) | Muris et al. (2017) |
| Notes. NPI = Narcissistic Personality Inventory, SRP = Self-Report Psychopathy Scale, Narc = Narcissism, Mach = Machiavellianism, Psych = Psychopathy. The range of reported correlations is shown in parentheses. Only statistically significant correlations greater than *r =*.10 are included. If multiple correlates are traced back to the same references, the references are only given for the last correlate. | | | |
