# Supplementary material for: Measuring the dark triad: a meta-analytical SEM study of two prominent short scales
Source: Front Psychol. 2025 Jan 15;15:1469970. doi: 10.3389/fpsyg.2024.1469970 (PMC11774945; doi:10.3389/fpsyg.2024.1469970)
Supplement: Supplementary file 2 [file Table_2.docx]

Supplementary Table 2. Formula Expressions of Reliability Coefficients for Different Measurement Models.

| Correlated factors model | | | |
| --- | --- | --- | --- |
| $\rho_{CF}$ | $\frac{\sum_{p=1}^{m} \sum_{q=1}^{m} \phi_{pq}\left( \sum_{i=1}^{k} \lambda_{ip} \right)\left( \sum_{i=1}^{k} \lambda_{iq} \right)}{\sigma_{x}^{2}}$ |  |  |
| ${}_{p}\rho_{CF}$ | $\frac{\left( \Sigma_{i\epsilon G_{p}}\lambda_{ip} \right)^{2}}{\sigma_{x_{i}\epsilon G_{p}}^{2}}$ |  |  |
| Bifactor Models | | | |
|  |  | % General Factor | % Group Factor |
| $\rho_{BF}$ | $\frac{\left( \sum_{i=1}^{k} \lambda_{iF} \right)^{2}+\sum_{p=1}^{m} \left( \sum_{i=1}^{k} \lambda_{ip} \right)^{2}}{\sigma_{x}^{2}}$ |  |  |
| $\rho_{BFS-1}$ | $\frac{\left( \sum_{i=1}^{k} \lambda_{iF} \right)^{2}+\sum_{p=1}^{m} \sum_{q=1}^{m} \phi_{pq}\left( \sum_{i=1}^{k} \lambda_{ip} \right)\left( \sum_{i=1}^{k} \lambda_{iq} \right)}{\sigma_{x}^{2}}$ |  |  |
| ${}_{p}\rho_{BF}$ | $\frac{\left( \sum_{i\epsilon G_{p}} \lambda_{iF} \right)^{2}+\left( \sum_{i\epsilon G_{p}} \lambda_{ip} \right)^{2}}{\sigma_{x_{i}\epsilon G_{p}}^{2}}$ | $\frac{\left( \sum_{i\epsilon G_{p}} \lambda_{iF} \right)^{2}}{\sigma_{x_{i}\epsilon G_{p}}^{2}}$ | $\frac{\left( \sum_{i\epsilon G_{p}} \lambda_{ip} \right)^{2}}{\sigma_{x_{i}\epsilon G_{p}}^{2}}$ |
| $\omega_{HBF}$ | $\frac{\left( \sum_{i=1}^{k} \lambda_{iF} \right)^{2}}{\sigma_{x}^{2}}$ |  |  |
| Notes. ρ with subscript is total reliability and quantifies how much variance all factors explain of the total model-implied variance. $\rho_{BF}$ are the subtest reliabilities of bifactor models and quantifies how much variance all factors of a subscale explain of the model-implied variance of the subscale. This is further divided into general and facet-specific factor components. Formulas for this are given in separate columns. $\omega_{HBF}$ is hierarchical omega of the general or general reference factor, which quantifies the variance it explains out of the total variance implied by the model. $\phi_{pq}$ is the correlation between the subscales *p* and *q*, where *p≠ q*. $\lambda_{iF}$ is the general factor loading of item *i*. $\lambda_{ip}$ / $\lambda_{iq}$ is the factor loading of item *i* on *p* / *q* the subscale G construct. *k* is the number of total items. *m* is the number of facet-specific factors. $\sigma_{x}^{2}$ is the total model-implied variance. $\sigma_{x_{i}\epsilon G_{p}}^{2}$ is the model-implied variance of the subscale $G_{p}$. All formulae, except $\rho_{BFS-1}$, have been taken from Cho (2016). | | | |
